# Oritavancin for the treatment of complicated gram-positive infection in persons who inject drugs

**DOI:** 10.1186/s40360-020-00452-z

**Published:** 2020-10-28

**Authors:** Aileen Ahiskali, Heather Rhodes

**Affiliations:** 1 Department of Pharmacy, Hennepin Healthcare, 701 Park Avenue, Minneapolis, MN 55415 USA; 2 Clinical Quality Improvement, Hennepin Healthcare, Orange 1.220-7, 701 Park Avenue, Minneapolis, MN 55415 USA

## Abstract

**Background:**

Treatment of complicated infections in persons who inject drugs (PWID) and patients experiencing homelessness poses a unique challenge to clinicians. Long-acting lipoglycopeptide antibiotics, such as oritavancin, may facilitate extended courses of outpatient intravenous therapy while avoiding the need for central lines, improving compliance and thus increasing the chance of clinical cure.

**Methods:**

Retrospective chart review of adult PWID who received at least one dose of oritavancin for a gram-positive infection between 1/1/17 and 6/30/19 at a large safety net hospital.

**Results:**

Twenty three PWID received 24 courses of at least one dose of oritavancin for a gram-positive infection; 16 were experiencing homelessness at the time of diagnosis. Methicillin resistant *Staphylococcus aureus* (MRSA) was the most common infecting pathogen and bone or joint the most frequent infection site. Nineteen encounters resulted in clinical cure, including 5 whose conditions improved despite non-adherence to their prescribed regimen. Three patients experienced a non-favorable outcome. Two patients experienced mild adverse drug reactions that did not interfere with therapy; no patients died while on therapy.

**Conclusion:**

Oritavancin may be a clinically effective treatment option for the management of complicated gram-positive infections in PWID and patients experiencing homelessness. Further studies should be performed to validate these results.

## Introduction

Effective strategies to improve serious infection treatment outcomes in non-adherent patients are lacking and complicated by injection drug use (IDU) and homelessness. Up to 70% of persons who inject drugs (PWID) experience at least 1 bacterial skin infection in their lifetime, but it is difficult to accurately describe the true breadth of the problem as a recent study found that more than half of all IDU-associated bacterial infections (ABIs) may be unrecorded [1–4]. Adding further complexity to treatment, the prevalence of homelessness among PWID has been reported to be as high as 59% [5]. Homelessness significantly increases the risk of relapse in those who have previously stopped injecting, promoting initial and recurrent IDU-ABIs [6].

Complex socioeconomic factors often limit the ability to administer first-line therapies. Outpatient parenteral antibiotic therapy (OPAT) is controversial due to concerns of inappropriate central line access and treatment failure risk [7]. Eaton et al. challenged this apprehension by employing a 9-point risk assessment to successfully administer OPAT to PWID, reducing average length of stay from 42 to 22 days [8]. However, after providers form trusting relationships with patients, home infusion services may still refuse to provide therapy to patients they deem high risk. When oral antibiotics are therapeutically appropriate, drug interactions (e.g. with rifampin), cost and repeated non-adherence may preclude use of this route. Thus, at times patients must remain admitted for multiple weeks to complete therapy, or leave against medical advice (AMA) and risk the potentially life-threatening consequences of inadequately treated infections. Additionally, prolonged admissions delay patient enrollment in outpatient rehabilitation programs, which in turn slows substance use disorder (SUD) treatment.

Oritavancin (ORI) is a long-acting lipoglycopeptide (LAL) that covers a broad range of gram-positive pathogens including methicillin-resistant and methicillin susceptible *Staphylococcus aureus* (MRSA, MSSA), *Streptococcus* species, and vanA-mediated vancomycin-resistant *Enterococcus* species and was FDA approved in 2014 for the treatment of acute bacterial skin and skin structure infections (ABSSSI) as a one-time 3 hour 1200 mg intravenous (IV) infusion [9]. Its long terminal half-life, large volume of distribution and penetration to bone and joint spaces make it appealing for treatment of deep seated infections that require IV therapy, but in whom this route may not be feasible in the outpatient setting. At our institution, ORI was selected as the formulary LAL due to patient assistance programs that were more relevant to our patient population and a lack of evidence demonstrating clinical superiority of dalbavancin (DAL), the other LAL, over oritavancin.

Hennepin County Medical Center (HCMC) is the largest safety net hospital in the state of Minnesota and a Level 1 Trauma and Burn destination caring for patients across the upper Midwest. Providers at our 484 bed institution routinely face challenges providing IV therapy for patients afflicted with serious mental health issues, SUD and homelessness. In 2018, HCMC providers treated over 130 cases of *Staphylococcus aureus* bacteremia, of which approximately one-third were attributed to IDU. Additionally, during 2018 alone, at least 10% of patients were readmitted with recurrent *S. aureus* bloodstream infections primarily due to non-adherence and/or reinfection. In an effort to provide adequate treatment courses, single- or multiple-dose regimens of ORI have been employed in select patients at our institution. We describe our clinical experience utilizing ORI for the treatment of complicated gram-positive infections in adult PWID, many of whom experienced homelessness.

## Methods

We performed a retrospective cohort analysis of PWID treated with ORI for gram-positive infection. Patients were included if they were 18 years of age or older and received at least one dose of ORI for the treatment of documented or presumed gram-positive endocarditis, bone/joint infection, bacteremia, or skin and soft tissue infection between 1/1/17 and 6/30/19. For patients who received more than one course of oritavancin during the study period, each course was assessed separately for inclusion. All patients in this study were evaluated by an infectious diseases (ID) physician as prescription of ORI is restricted to ID physicians at our institution due to its high drug acquisition cost and broad gram-positive spectrum of activity; pharmacy does not release ORI unless authorized by ID. Infection indication was identified by the ID consult note. Susceptibility testing for ORI was not performed on any isolates, but was inferred from vancomycin susceptibility based on previous studies’ findings [10]. Patients who received concomitant antibiotics were included in the analysis; their additional therapy is included in Table 1. For multiple dose regimens, doses were administered once weekly until completed. Patients were excluded if more than 14 days elapsed between administered doses, their care was palliative in nature, they were pregnant or a prisoner at the time of treatment, or had declined use of their information for research purposes.

**Table 1:** Antibiotic course and clinical outcomes of patients who inject drugs treated with oritavancin

#	Sex, Age range, BMI	Additional Social History	Infection Location, Source Control Achieved?	Pathogen(s)	Gram- Positive Antibiotics Prior to ORI (days)	Abx DOT before ORI	Concurrent antibiotics with ORI	ORI Doses Planned (received)	Outcome
1	M, 40–49, 22.9	Homeless	BSI; SSTI Yes	MRSA	VAN × 8	8	None	1200 × 1 (1)	CC
2	M, 40–49, 30.1	Homeless	Joint Yes	MRSA + Enterobacter	VAN × 1	1	LVX - Enterobacter	1200 × 2 (2)	CC
3	M, 40–49, 20.9	Homeless ETOH	SSTI N/A	Unknown	None	0	None	1200 × 1 (1)	CC
4	F, 40–49, 23.6	Homeless ETOH	SSTI N/A	MRSA	VAN × 2	2	None	1200 × 1 (1)	CC
5	M, 30–39, 24.5	ETOH	Joint Yes	MSSA; GAS	CRO × 2 VAN × 1	2	None	1200 × 1 (1)	CC
6	F, 50–59, 39.5	Homeless ETOH	BSI; SSTI Yes	MSSA	VAN + TZP × 2 NAF × 3 CFZ × 22	25	None	1200 × 2 (1)	I/C
7	F, 40–49, 19.6	Homeless ETOH	BSI; Bone Yes	MSSA	LVX × 1 VAN + TZP × 2 CFZ × 24	25	None	1200 × 2 (2)	CC
8	M, 40–49, 22.8	Homeless	BSI; Bone; SSTI No	MRSA	VAN × 6 DAL × 1	13	None	1200 × 1 (1)	CC
9	M, 40–49, 32.2	Homeless	Joint; HW Yes	Strep mitis; Strep oralis	TZP × 3 VAN × 4 CRO × 8 LVX × 8–17	36	None	1200 × 1 (1)	CC
10	F, 30–39, 26.5		BSI; IE No	MSSA	VAN + TZP × 2 NAF × 2 CFZ × 16–23 VAN + TZP × 2 CFZ × 6	30	None	1200 × 1 (1)	CC
11	M, 50–59, 26.0		BSI; IE; Bone No	MRSA	VAN × 9 CPT + DAP × 14 *none × 4 days* CPT × 5 DAP × 22	47	None	1200 × 2 (2)	F
12	F, 30–39, 32.5	Homeless	BSI N/A	MSSA	CRO × 2 VAN × 3 CFZ × 15 None × 2 CFZ × 2	20	None	1200 × 1 (1)	CC
13	M, 40–49, 34.1	Homeless	BSI N/A	MSSA	VAN × 3 CFZ × 7 CRO × 1	10	None	1200 × 1 (1)	LTFU
14	M, 30–39, 28.9	Homeless ETOH	BSI; SSTI N/A	MRSA	VAN × 15 DAP × 1	16	None	1200 × 2 (2)	LTFU
15	M, 60–69, 44.6		Joint; HW No	MRSA, MRSE	VAN × 6 DOX × 5 VAN × 4	13	None	1200 × 1 (1) 800 × 5 (5)	CC
16	F, 20–29, 24.2	Homeless ETOH	Bone No	MRSA	VAN × 1	1	None	1200 × 2 (2)	I/C
17	M, 20–29, 22.1	Homeless	Bone; HW No	MSSA	VAN + TZP × 2 CFZ × 2	3	RIF	1200 × 2 (2)	CC
18	M, 30–39, 19.6	Homeless ETOH	Bone; SSTI Yes	MRSA	VAN × 5 DOX × 2	7	DOX	1200 × 2 (1)	I/C
19	F, 30–39, 36.2		BSI; Bone No	MRSA	VAN × 9	9	None	1200 × 4 (3)	I/C
20	F, 20–29, 23.0		Bone No	MRSA	VAN × 13	13	None	1200 × 1 (1) 800 × 3 (1)	I/C
21	M, 20–29, 23.4		BSI; SSTI N/A	MSSA	VAN × 2 CFZ × 4	4	None	1200 × 2 (1)	I/F
22	M, 40–49, 32.2	Homeless	Joint Yes	MRSA	VAN × 2	2	RIF	1200 × 1 (1) 800 × 5 (5)	CC
23	M, 30–39, 22.8	ETOH	BSI N/A	MRSA	VAN × 6	6	None	1200 × 1 (1)	F
24	M, 60–69, 18.0	Homeless	Bone No	MRSA	DAP × 4	4	None	1200 × 1 (1) 800 × 3 (3)	CC

*Heading abbreviations: Abx* antibiotic, *BMI* body mass index, *DOT* days of therapy

*Social history abbreviations:* *ETOH* ethanol

*Infection location abbreviations: BSI* bloodstream infection, *HW* hardware, *IE* infective endocarditis, *SSTI* skin and soft tissue infection

*Pathogen abbreviations: GAS* group A strep, *MRSA* methicillin resistant staph aureus, *MSSA* methicillin susceptible staph aureus, *MRSE* methicillin resistant staph epidermidis

*Antimicrobial abbreviations:CFZ* cefazolin, *CRO* ceftriaxone, *FEP* cefepime, *CPT* ceftaroline, *DAL* dalbavancin, *DAP* daptomycin, *DOX* doxycycline, *LVX* levofloxacin, *LZD* linezolid, *MTZ* metronidazole, *MEM* meropenem, *NAF* nafcillin, *ORI* oritavancin, *TZP* piperacillin-tazobactam, *RIF* rifampin, *SXT* trimethoprim-sulfamethoxazole, *VAN* vancomycin

*Treatment evaluation outcomes: CC* clinical cure, *F* failure, *I/C* incomplete (patient didn’t adhere to intended regimen but appears to have been cured), *I/F* incomplete adherence and clinical failure, *LTFU* lost to follow up

Both authors independently reviewed each case and made a determination of outcome. Results were categorized as clinical cure or failure based on manual chart review. Outcomes were further described as incomplete adherence/cure (I/C) or incomplete adherence/failure (I/F) if a dose was missed based on the initially planned regimen. Clinical cure was defined as resolution of signs and symptoms of infection (fever, white blood cell count, C-reactive protein) without need for additional antimicrobial therapy following completion of ORI, excluding long term suppressive antibiotics for patients with retained hardware. Failure was defined as progression of gram-positive infection and need for alternative therapy. Outcomes were reviewed out to 60 days after the final ORI infusion.

Adverse drug reactions (ADRs) were collected up to 6 weeks from the last dose or up to the point that a patient was lost to follow up. All notes available in the institution’s electronic medical record (EMR) were examined for mention and description of ADRs. Outside hospital records were reviewed if available at the time of review by way of Epic’s Care Everywhere. The Naranjo Adverse Drug Reaction Probability Scale (NADRPS) was employed to determine the probability the ADR was attributable to oritavancin [11].

This study was reviewed and approved by the Human Subjects Research Committee of the Hennepin Healthcare Research Institute.

## Results

A list of all 37 encounters in which a patient received one or more doses of oritavancin within the specified time frame supplied the starting point for review. Thirteen encounters were excluded; the primary reason for exclusion was no known history of injection drug use (*n* = 9); other reasons included a diagnosis outside of the inclusion criteria (*n* = 2) and greater than 14 days elapsed between administered doses (*n* = 2). Twenty four courses, prescribed to 23 different patients, were included in the analysis. One patient (numbers 2 and 22 in Table 1) received 2 separate courses of treatment, with the second course administered 20 months after the final dose of the initial course. At the time of infection diagnosis, 16/24 (67%) encounters were for patients experiencing homelessness. Most patients were male (16/23, 70%) and the average age was 41 years old (range 22–64). The median body mass index (BMI) was 24.3; 8/24 (33%) were considered obese with a BMI of greater than 30. Sixteen of 23 patients (70%) had a history of significant psychiatric illness defined as schizophrenia, bipolar disorder, major depressive disorder, schizoaffective disorder or borderline personality disorder. Nine (39%) patients left AMA during a prior hospitalization for their infection.

MRSA was the most common infecting pathogen occurring in 14/24 (58%) of encounters. Bone or joint was the most frequent infection location occurring in 14/24 (58%) encounters, and the spine was the most common site of bone infection (4/9, 44%). Half of all encounter patients (12/24, 50%) were bacteremic with either MRSA (6/12, 50%) or MSSA (6/12, 50%). Of the two patients diagnosed with infective endocarditis, both involved native tricuspid valves and neither underwent surgical valve replacement.

Patients received a median of 9.5 days of effective gram-positive therapy based on susceptibility, when available, prior to ORI initiation. Number of ORI doses ranged from 1 to 6; initial doses were universally 1200 mg. Subsequent dosing was at the discretion of the clinician and was always administered weekly. Eleven patients received more than one dose. Of those, 4 received 800 mg doses and 7 received 1200 mg doses. The majority of doses were administered in the outpatient infusion center. Twelve patients received 1 dose as inpatient; 3 of these patients received additional outpatient doses thereafter. Twenty three of 24 encounters utilized ORI for therapy completion, having received prior gram-positive treatment for their infections. Patient 8 was the only patient to also receive DAL; the patient received a single dose at an outside hospital prior to transferring care to our institution.

Clinical cure was achieved in 19/24 (79%) encounters and failure in 3/24 (13%); 2/24 (8%) were lost to follow-up after their last infusion. The two patients lost to follow-up were not noted to have any signs of worsening of infection at their final infusion, however, documentation was limited at each of those visits and thus no assessments can be made. Of the 4 patients with osteomyelitis involving the spine, 3/4 (75%) experienced clinical cure and 1/4 (25%) treatment failure. Of the 6 patients who had incomplete adherence to the planned regimen, cure was seen in 5 patients and failure in 1. Three patients received only the first of two planned doses; including the patient deemed to be a clinical failure in the group with partial adherence. The remaining 3 patients with partial adherence experienced an unexpected delay of 14 days between doses, but received all planned doses (3 to 5 doses total).

Two patients (8%) experienced ADRs within 6 weeks of receiving ORI. One patient presented to the Emergency Department (ED) 5 days after receiving ORI complaining of sharp, non-radiating abdominal pain, however, the patient eloped prior to evaluation. Four days after her ED visit she was admitted to the jail medical ward, which is overseen by our institution, without documentation of infection, pain or further antibiotics. One patient experienced an infusion-related reaction becoming visibly flushed and complaining of a headache 20 min into the first dose. After an infusion pause, the remaining drug was administered without symptom recurrence and she received a second dose 1 week later without issue. Employing the NADRPS harm scale, the former patient’s ADR was classified as possible and the latter as probable.

Three patients (patients 11, 21, and 23) were determined to have failed therapy with ORI, with 1 of 3 possibly related to incomplete adherence. Patient 11 was being treated for MRSA ((vancomycin minimum inhibitory concentration (MIC) 2, confirmed by two methods, Vitek and Microscan)) bacteremia with vertebral osteomyelitis and was initially bacteremic for 14 consecutive days. Source control was unable to be achieved due to location of fluid collections and proximity to spinal cord. After blood culture clearance, he was discharged and returned for daily infusions of high-dose daptomycin (> 10 mg/kg) but was transitioned to weekly ORI 3 weeks later due to loss of IV access and inability to receive a peripherally inserted central catheter (PICC). After the second ORI infusion, the patient was readmitted due to ongoing back pain and new spinal cultures were obtained; daptomycin MIC had increased from 0.5 to 4 μg/mL and thus was no longer susceptible. Although the corresponding vancomycin MIC of this isolate was 1 μg/mL, the ID team did not treat with vancomycin due to recent history of MRSA isolate with vancomycin MIC 2 μg/mL and the concern for emergence of resistant subpopulations. At the discretion of the ID physician, the patient remained admitted to complete 6 weeks of ceftaroline.

Patient 21 was treated for MRSA bacteremia and ABSSSI and missed the second of two planned ORI doses. He was admitted 14 days after his ORI infusion (7 days after the missed dose) and found to have recurrent MRSA bacteremia along with a new aortic valve vegetation on transthoracic echocardiography (TTE) that was not seen on TTE during his last admission.

Patient 23 is a paraplegic man with no sensation in his lower extremities, initially treated with IV vancomycin for MRSA bacteremia. On day 7 he planned to leave AMA; oral antibiotics could not be prescribed due to drug interactions so he was given a single ORI dose. The patient was readmitted for inpatient psychiatry care a few weeks later where he experienced a femur fracture while adjusting himself in bed. He was taken to surgery; intraoperative cultures grew MRSA but blood cultures remained negative. Both MRSA isolates, from the pre-ORI blood cultures and post-ORI intraoperative cultures, had a vancomycin MIC of 1 μg/mL via Vitek.

## Discussion

We describe our real-world experience using ORI for the treatment of complicated gram-positive infections in PWID, many of whom were also experiencing homelessness. Despite having limited treatment options for patients that are noncompliant with oral therapies or otherwise not candidates to receive IV antibiotics, alternative therapeutic regimens are often discredited or avoided due to a lack of robust clinical evidence. Although more data are emerging regarding LAL use for complicated infections, studies specific to ORI use in vulnerable populations remain limited.

When comparing our study to others that included outcomes specific to persons who use drugs (PWUD), it is important to note that each investigation evaluated outcomes differently. In an effort to compare findings despite methodological differences, data specific to PWUD in similar studies are outlined in Table 2. Overall, our rates of clinical cure (79%) and failure (13%) were similar to other reports [12–16].

**Table 2:** Comparison of long acting lipoglycopeptide studies that included persons who use drugs

Citation, year, LAL(s)	Patients	Organisms	Indications	Number of doses	Results	Outcomes & Definitions
Stewart et al. 2017 [12] ORI	▪ **Total**: 10 ▪ PWID: 4 (40%) **PWID only** ▪ Males: 0 ▪ Average age: 37 years (range 26–53)	**PWID** ▪ MSSA: 2 ▪ GBS: 1 ▪ Enterococci: 1	**PWID** ▪ GBS BSI + IE ▪ *E. faecalis* BSI ▪ BSI + bone/joint ▪ BSI + psoas abscess	**PWID** ▪ 1 dose = 4/4	**PWID** (*n* = 4) ▪ Cure: 1/4 ▪ Failure: 2/4 ▪ LTFU: 1/4	▪ **Clinical cure**: resolution of all clinical signs/symptoms of infection (afebrile, normalization of WBC/ESR), no additional infection-related hospital admissions, no additional antibiotic therapy required for the initial indication treated with ORI, infection clearance with sterile blood cultures where indicated. ▪ **Failure**: worsening of current infection or new/recurrent signs or symptoms of infection requiring a change in antibiotics or additional antibiotic therapy. ▪ **Not clinically evaluable**: patients who were LTFU. ▪ **ADRs**: based on reporting by prescribing physician and follow-up with patients after administration (timeframe not specified).
Morrisette et al. 2019 [14] DAL + ORI	▪ **Total:** 56 ▪ PWUD: 17 (30%) **PWUD only** ▪ Males: 12 (71%) ▪ Average age: 34.5 years (SD + 10.9) ▪ DAL: 12 /17 ▪ ORI: 4/17 ▪ Both: 1/17	**PWUD** ▪ MSSA: 8 (47%) ▪ MRSA: 5 (29%) ▪ *E. faecalis*: 1 (6%) ▪ VRE: 1 (6%) ▪ Other: 2 (12%) *(unknown or mixed)*	**PWUD** ▪ ABSSSI: 6 (35%) ▪ OM: 6 (35%) ▪ IE: 3 (18%) ▪ Other: 2 (12%)	**PWUD** ▪ Median = 1 (IQR 1–2)	**PWUD** (*n* = 17) ▪ Success: 13/17 (77%) ▪ Failure: 1/17 (6%) ▪ Unknown: 3/17 *1 patient counted as both a failure and success as they failed oritavancin but were subsequently cured with dalbavancin.*	▪ **Clinical success**: no further clinical or microbiological evidence of active infection (resolution of signs/symptoms related to bacterial infection and clearance of cultures if applicable) without need for further gram-positive therapy due to clinical worsening within 60 days of last dose of LAL. ▪ **Clinical failure**: lack of clinical response, relapse with the primary infection within 60 days of last LAL dose, need for alternative gram-positive therapy due to clinical worsening during LAL therapy, or death. ▪ **ADRs**: any potential ADR occurring during or within 7 days of LAL infusion.
Bork et al. 2019 [15] DAL	▪ **Total:** 28 ▪ PWUD: 19 (68%) ▪ PWID: 16/19 (57%) **All patients** ▪ Males: 26 (93%) ▪ Median age: 52 years (IQR 21.5)	**All patients** ▪ MRSA: 8 (29%) ▪ MSSA: 6 (21%) ▪ CoNS: 4 (14%) ▪ Mixed GP: 8 (29%) ▪ Unknown: 5 (18%)	**All patients** ▪ Bone: 13 (46%) ▪ Endovascular: 6 (21%) ▪ BSI: 4 (14%) ▪ Other: 5 (18%)	**All patients** ▪ Average = 3 ▪ Site 1: 1.5 ▪ Site 2: 5.5	**Substance Abusers** (*n* = 19) ▪ Success: 10/13 (77%) ▪ Failure: 3/13 (23%) ▪ LTFU: 6/19	▪ **Primary outcome**: proportion of patients with 30 day clinical cure (30 days after planned DAL course). ▪ **Secondary outcomes**: 90 day clinical cure; ADRs ▪ **Clinical cure**: composite of resolution of clinical signs of infection (erythema, swelling, pain); afebrile; normalization of CRP/ESR/WBC; source control; resolution of radiographic signs of infection and/or microbiologic clearance of organisms. If readmitted for infection then considered failure. ▪ **Clinical failure**: due to (1) death, (2) intolerance or AE, (3) lack of access to subsequent DAL, (4) lack of source control, (5) worsening signs of infection or relapse. ▪ **ADRs**: anything likely to be associated w/ DAL based on specific ADE and temporal relationship with DAL (specific timeframe not provided).
Bryson-Cahn et al. 2019 [16] DAL	▪ **Total**: 32 ▪ PWUD: 32 (100%) ▪ PWID: 28 (88%) ▪ Homeless: 15 (47%) **All patients (PWUD)** ▪ Males: 23 (72%) ▪ Average age: 38 years (IQR 25–50)	**PWUD** ▪ MRSA: 28 (88%) ▪ In the IE group: -MSSA = 2 -MRSA = 7 *Other organisms not reported.*	**PWUD** ▪ IE: 9 ▪ BSI: 14 - Joint: 2 - Epidural abscess: 3 - Thrombophlebitis: 2 ▪ Bone: 7 - Spine: 3 - Extremity: 4 ▪ Joint: 2 *(without BSI)*	**PWUD** ▪ 1 dose = 22 ▪ 2 doses = 7 ▪ 3 doses = 2 ▪ 5 doses = 1	**PWUD** (*n* = 32) ▪ Success: 18/32 (56%) ▪ Failure: 4/32 (13%) ▪ LTFU: 10/32 (31%) ▪ Completed therapy: 17/32 (53%)	▪ **Clinical response**: patient had a follow-up visit within 1 year at HMC or a neighboring hospital, linked through a common EMR, without evidence for an ongoing or relapsed infection, regardless of whether they completed the intended course of therapy. ▪ **LTFU**: patient did not have a subsequent encounter to evaluate their infection at either the ID clinic or another institution linked via EMR within 1 year. ▪ **All-cause readmission**: hospital readmission within 30 days from previous discharge. ▪ **ADRs**: not defined/reported.
Brownell et al. 2020 [13] ORI	▪ **Total:** ▪ 75 safety analysis ▪ 73 efficacy analysis ▪ PWID: 10 (13.3%)	**All patients:** ▪ MSSA: 23 (31.5%) ▪ MRSA: 13 (17.8%) ▪ CoNS: 4 (5.8%) ▪ VRE: 4 (5.8%)	**All patients:** ABSSSI: 25 Diabetic foot infection: 3 IE: 4 Line infection: 2 Other: 5 OM/Septic arthritis: 10 Pneumonia: 5 Prosthetic device infection: 3 Sepsis: 5 Surgical wound infection: 12 **PWID: numbers not reported** OM, IE, Prosthetic device infection, Surgical wound infection	**All patients:** 1–32	**All patients:** ***n*** **= 73** Cure: 34/73 (46.6%) Improvement: 34/73 (46.6%) Failure: 5/73 (6.8%) **PWID (*n*** **= 10)** Cure or improvement: 7/10 (70%)	• **Clinical outcomes **were reviewed within 28 days of the end of therapy. Safety outcomes were reviewed out to 30 days after ORI. • **Clinical cure**: resolution of signs and symptoms of infection without the need for further treatment after the completion of ORI • **Clinical improvement**: recovery from infectious signs and symptoms with the need for subsequent gram-positive therapy after the completion of the ORI • **Clinical failure**: progression of infectious signs and symptoms and need for alternative gram-positive therapy during the ORI course
Ahiskali et al. 2020 ORI	▪ **Total** = 24 ▪ PWUD: 24 (100%) ▪ PWID: 24 (100%) ▪ Homeless: 16 (67%) **All patients (PWUD)** ▪ Males: 16 (70%) ▪ Average age: 41 years (range 22–64)	**PWUD** ▪ MRSA: 14 (58%) ▪ MSSA: 8 (33%) ▪ Streptococci: 2 ▪ Unknown: 1	**PWUD** ▪ Bone/Joint: 14 - Bone: 9 *Spine: 5/9* - Joint: 5 ▪ BSI: 12 ▪ IE: 2 *Patients may be counted in multiple categories.*	**PWUD** ▪ 1 dose = 13 ▪ 2 doses = 7 ▪ 3 doses = 1 ▪ 4 doses = 1 ▪ 6 doses = 2	**PWUD** (*n* = 24) ▪ Success: 19/24 (79%) - I/C = 5 ▪ Failure: 3/24 (13%) - I/F = 1 ▪ LTFU: 2/24 (8%) ▪ Completed therapy: 19/24 (79%)	▪ Outcomes were reviewed out to 60 days after the final ORI infusion. ▪ **Clinical cure**: resolution of signs and symptoms of infection (fever, WBC, ESR) without need for additional antimicrobial therapy following completion of ORI (excluding long term suppressive antibiotics for retained hardware). ▪ **Failure**: progression of infection and need for alternative therapy. ▪ **Incomplete cure/failure**: incomplete adherence to therapy. ▪ **LTFU**: no notes or data available in EMR after final ORI dose. ▪ **ADRs**: collected up to 6 weeks from last dose or until LTFU.

*Abbreviations:* ABSSSI acute bacterial skin and skin structure infection, *BSI* bloodstream infection, *CC* clinical cure, *CoNS* coagulase-negative staphylococci, *DAL* dalbavancin, *EMR* electronic medical record, *ESR* erythrocyte sedimentation rate, *GBS* Group B Streptococcus, *GP* gram-positive, *HW* hardware, *IE* infective endocarditis, *I/C* incomplete adherence with cure, *I/F* incomplete adherence with failure, , *IQR* interquartile range, *LTFU* lost to follow-up, *MSSA* methicillin-susceptible *Staphylococcus aureus*, *MRSA* methicillin-resistant *Staphylococcus aureus*, *OM* osteomyelitis, *ORI* oritavancin, *PWID* persons who inject drugs, *PWUD* persons who use drugs, *VRE* vancomycin-resistant *Enterococcus faecium*, *WBC* white blood cell count

A comparable study focused on complicated infections in vulnerable patients treated with LALs but outcomes were specific to DAL [16]. Although ORI and DAL are similar, it is important to highlight their differences to further appreciate our study’s contribution to existing literature. While both boast an exceptionally long half-life (t½) based on population pharmacokinetic analyses, ORI’s terminal t½ is slightly longer, 10.2 vs. 8.5 days and the plasma protein binding of DAL is 93% as compared to 85% with ORI. Oritavancin has a large volume of distribution (Vd) (1.25 L/kg) indicating extensive tissue distribution, whereas DAL’s smaller Vd (0.11 L/kg) indicates it primarily remains in the plasma compartment [9, 17]. Although we acknowledge the presence of these and other differences, the clinical impact is unclear as both drugs have been associated with successful outcomes in the treatment of complicated infections [12, 13, 14–16]. In addition, clinical scenarios exist where DAL may be preferred, such as for genitourinary sources as < 5% of ORI is recovered unchanged in the urine compared to 33% of DAL [9, 17].

Our study is not without limitations. All but one patient received ORI as secondary therapy and all bacteremic patients received the drug following clearance of blood cultures, so no determination may be made regarding the utility of this agent for primary therapy. Patients received varying regimens for similar indications and thus it is not possible to evaluate a specific regimen for a given indication. Finally, generalizability may be limited due to the single center design of the study.

## Conclusion

In conclusion, we report our real-world experience using ORI in PWID primarily for treatment completion of complicated gram-positive infections. We acknowledge the delicate balance between antimicrobial stewardship efforts and financial considerations in providing a high cost, broad spectrum agent to ensure patients receive a safe, effective regimen that avoids extended hospitalizations and incomplete treatment courses. We believe ORI may be considered in patients who require prolonged therapy courses but are unable to receive OPAT, including PWID and patients experiencing homelessness. Randomized controlled studies should be conducted to determine optimal dosing regimens for off-label indications and to compare LAL therapy to standard of care.

## Data Availability

All data analyzed during this study are included in this published article and may be found in Table 1.

## Abbreviations

PWID: Persons who inject drugs
MRSA: Methicillin resistant *Staphylococcus aureus*
IDU: Injection drug use
ABIs: Associated bacterial infections
OPAT: Outpatient parenteral antibiotic therapy
AMA: Against medical advice
SUD: Substance use disorder
ORI: Oritavancin
LAL: Long-acting lipoglycopeptide
MSSA: Methicillin susceptible *Staphylococcus aureus*
ABSSSI: Acute bacterial skin and skin structure infections
IV: Intravenous
DAL: Dalbavancin
HCMC: Hennepin County Medical Center
ID: Infectious diseases
I/C: Incomplete adherence/cure
I/F: Incomplete adherence/failure
ADRs: Adverse drug reactions
EMR: Electronic medical record
NADRPS: Naranjo Adverse Drug Reaction Probability Scale
BMI: Body mass index
ED: Emergency Department
MIC: Minimum inhibitory concentration
PICC: Peripherally inserted central catheter
TTE: Transthoracic echocardiography
PWUD: Persons who use drugs
t½: Half-life
Vd: Volume of distribution
